# Comparative Sleep Architecture Profiles of Antidepressants in Mice: Pharmacological Characterization of Paroxetine, Sertraline, Duloxetine, Mirtazapine and Vortioxetine

**DOI:** 10.1002/prp2.70246

**Published:** 2026-05-04

**Authors:** Junya Maruoka, Ryo Egami, Yuto Akita, Kohei Kozuka, Yusuke Kakumoto, Tetsuro Kikuchi, Kazuhiko Kume

**Affiliations:** ^1^ Tokushima Research Center for Drug Discovery Otsuka Pharmaceutical Co., Ltd. Tokushima Tokushima Japan; ^2^ Department of Neuropharmacology, Graduate School of Pharmaceutical Sciences Nagoya City University Nagoya Aichi Japan; ^3^ Department of Neuropharmacology, Faculty of Pharmaceutical Sciences Nagoya City University Nagoya Aichi Japan

**Keywords:** antidepressant, duloxetine, mirtazapine, paroxetine, sertraline, sleep architecture, vortioxetine

## Abstract

Sleep is a biological process essential for various brain functions, and patients with major depressive disorder (MDD) frequently experience sleep disturbances. Since such disturbances are both symptoms and risk factors for depression, evaluating the effects of antidepressants on sleep is important for understanding their clinical effectiveness. In this study, we investigated the effects of five antidepressants—paroxetine and sertraline (selective serotonin reuptake inhibitors), duloxetine (a serotonin‐norepinephrine reuptake inhibitor), mirtazapine (a noradrenergic and specific serotonergic antidepressant), and vortioxetine (a serotonin reuptake inhibitor and modulator)—on sleep architecture in male mice using cortical electroencephalogram and electromyogram. Paroxetine and sertraline reduced rapid eye movement (REM) sleep, while duloxetine decreased REM sleep and increased non‐REM (NREM) sleep. Mirtazapine reduced Wake and increased NREM sleep significantly, while vortioxetine also increased NREM sleep. Interestingly, among the five antidepressants tested, only paroxetine reduced Wake and increased NREM sleep during the dark period when administered prior to the light period. This is the first study to compare the effects of these five compounds on sleep architecture in mice within a single experimental framework. These findings were consistent with clinical observations, suggesting translational relevance. By reporting and comparing the sleep‐related pharmacological profiles of these antidepressants, our findings may help guide the selection of appropriate treatments for patients with MDD, particularly those suffering from sleep disturbances.

## Graphical abstract

This study compared five antidepressants (paroxetine, sertraline, duloxetine, mirtazapine, and vortioxetine) in mice using EEG/EMG, revealing distinct effects on REM and NREM sleep. The figure illustrates changes in sleep architecture during the light phase (ZT0–12). These pharmacological profiles align with clinical observations and may guide antidepressant selection for patients with major depressive disorder experiencing sleep disturbances.

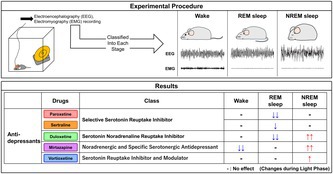

**Abbreviations**
5‐HT: 5‐hydroxytryptamine
AUC: area under the curve
EEG: electroencephalogram
EMG: electromyogram
MDD: major depressive disorder
NA: noradrenaline
NaSSA: noradrenergic and specific serotonergic antidepressant
NREM: non‐REM
REM: rapid eye movement
SNRI: serotonin‐norepinephrine reuptake inhibitor
S‐RIM: serotonin reuptake inhibitor and modulator
SSRI: selective serotonin reuptake inhibitor
ZT: zeitgeber time

## 1 Introduction

Sleep is a fundamental biological process conserved across a wide range of species [1]. During sleep, critical functions such as memory consolidation, neural and physical restoration, vigilant attention, and the clearance of metabolic waste products take place [2, 3, 4, 5]. Moreover, sleep disturbances are commonly observed among individuals with psychiatric disorders [2, 6].

Major depressive disorder (MDD) is a highly prevalent and debilitating psychiatric condition, affecting approximately 300 million individuals globally [7]. MDD is characterized by depressive mood, anhedonia, decreased interest in pleasurable activities, poor concentration, sleep disturbance, and irritability [8]. Approximately 90% of individuals with MDD experience sleep disturbance [9, 10, 11]. Patients with MDD exhibit significant abnormalities in sleep architecture, such as prolonged sleep latency, increased wake after sleep onset, reduced sleep efficiency, and reductions in slow‐wave sleep [12, 13]. Among the most robust biological markers of MDD are alterations in rapid eye movement (REM) sleep, including shortened REM latency, increased REM duration and density, and a greater proportion of REM sleep [10].

Various classes of antidepressants with distinct mechanisms of action have been developed. Starting with the first developed tricyclic antidepressants and monoamine oxidase inhibitors, it was discovered that neurotransmitters such as 5‐hydroxytryptamine (5‐HT) and norepinephrine played an important role in improving MDD [14, 15, 16]. Subsequently, several types of drugs were developed, including selective serotonin reuptake inhibitors (SSRIs), serotonin‐norepinephrine reuptake inhibitors (SNRIs), and noradrenergic and specific serotonergic antidepressants (NaSSAs) that promote neurotransmitter release via adrenaline α_2_ receptors [17, 18, 19]. To enhance the therapeutic efficacy of SSRIs, a novel class of antidepressants known as serotonin reuptake inhibitor and modulator (a S‐RIM) has recently been developed. This agent not only inhibits 5‐HT reuptake but also modulates several 5‐HT receptors [20, 21, 22].

Despite the development of various classes of antidepressants with distinct mechanisms of action, sleep disturbances remain one of the most frequent residual symptoms in individuals receiving antidepressant therapy for MDD [23]. A recent review article reported that subjective complaints of insomnia and daytime somnolence are common among patients with depression or anxiety disorders being treated with SSRIs or SNRIs [24]. Given that persistent sleep disturbances are both a core symptom and a risk factor for MDD, understanding how antidepressants influence sleep is essential for optimizing treatment outcomes [25]. However, existing preclinical studies have been conducted under diverse experimental conditions, including differences in species, housing environments, and administration timing, making it difficult to reliably compare pharmacological profiles across studies. While consideration of sleep‐related effects is important in the treatment of MDD, no preclinical studies have been reported that compare the effects of antidepressants with various mechanisms of action on sleep architecture in mice. In this study, we investigated five antidepressants with distinct mechanisms of action—paroxetine and sertraline (SSRIs), duloxetine (SNRI), mirtazapine (NaSSA), and vortioxetine (S‐RIM)—using electroencephalogram (EEG) and electromyogram (EMG) recordings in male mice to systematically evaluate and compare their effects on sleep architecture. Male mice were used to minimize variability associated with the estrous cycle.

## 2 Materials and Methods

### 2.1 Ethical Considerations

All animal protocols were approved by the Nagoya City University (approval number: NCU‐YD21‐001). All experiments in this study have been carried out in accordance with the Guide for the Care and Use of Laboratory Animals as adopted and promulgated by the US National Institutes of Health, and all animals in this study were treated in accordance with the relevant Guidelines for Animal Care and Use at the Institutional Guidelines on Animal Experimentation of Nagoya City University.

### 2.2 Animals

Male C57BL/6J mice (14–22 g, 5 weeks old, Clea Japan Inc., Japan) were purchased. Upon arrival, mice were acclimatized to the animal facility for at least 1 week prior to surgery. Following surgery and a recovery period of at least 7 days, mice were habituated to the recording apparatus for approximately 2 weeks. Consequently, all pharmacological experiments were conducted on mice that were at least 9 weeks old. A total of 45 mice underwent surgery; however, due to device failure, accurate recordings were obtained from 38 mice (*N* = 38). Different mice were used for the evaluation of each antidepressant; no animal was reused for testing different compounds. Mice were housed in a room maintained at 23°C ± 2°C with an alternating 12 h light–dark cycle. Food and water were available ad libitum.

### 2.3 Drugs

Based on a previous study, we prepared a solution (vehicle) by dissolving 18% gelatin, 0.58% sucralose, and 0.28% sodium carboxymethylcellulose in water [26]. Paroxetine hydrochloride (FUJIFILM Wako Pure Chemical Corporation, Japan), sertraline hydrochloride (Tokyo Chemical Industry Co. Ltd., Japan), duloxetine hydrochloride (FUJIFILM Wako Pure Chemical Corporation, Japan), mirtazapine (Sigma‐Aldrich Co. LLC, USA), and vortioxetine hydrobromide (MedChemExpress Co. Ltd., USA) were dissolved in the vehicle. The dosage of each drug was determined based on previous studies [27, 28, 29, 30, 31]. Drugs were administered via voluntary oral consumption, and each mouse consumed the vehicle or drugs at a volume of 10 mL/kg body weight without physical restraint or anesthesia. Zeitgeber time (ZT) 0 was defined as the onset of the light period (lights‐on). Consequently, the light period corresponded to ZT0–12, and the dark period to ZT12–24. All drug administrations were performed 30 min prior to ZT0.

### 2.4 Surgery (EEG/EMG Electrode Implantation)

Before the surgery, mice were habituated to a home cage equipped with a running wheel for more than 3 days. Mice were anesthetized by isoflurane and fixed using a stereotaxic apparatus (SM‐6 M‐HT, Narishige, Japan). For EEG recording, two stainless steel screw electrodes were placed on the cortex, while a third screw was placed on the cerebellum as the ground electrode. EEG electrodes were implanted at + 1.0 mm AP and + 1.5 mm ML from bregma or + 1.5 mm AP and + 1.5 mm ML from lambda [32, 33]. For EMG recording, two stainless steel wires were bilaterally inserted into the trapezius muscles. All electrodes were fixed to the skull using dental cement. After surgery, mice were separately housed during the recovery period for at least 7 days before recording. Mice exhibiting unstable EEG signals were excluded from the analysis. After the animals were euthanized, we confirmed the electrode placement by visual inspection in all mice.

### 2.5 EEG/EMG Recording

The mouse was placed in an acrylic box (30 cm × 30 cm × 30 cm) equipped with a running wheel, with free access to food and water. The mouse was habituated to this box for approximately 2 weeks. Next, EEGs and EMGs were obtained from mice able to move freely. EEG and EMG signals were obtained at a sampling rate of 100 Hz. These recorded data were processed with a bandpass filter in the range of 0–50 Hz and saved in digital format.

### 2.6 Data Analysis for EEG/EMG Study

EEG and EMG signals were analyzed using SleepSign software version 3 (Kissei Comtec, Japan). One epoch was defined as 10 s and each epoch was further classified into Wake, REM sleep or NREM sleep stage based on a previous study [34]. The Wake stage was determined primarily based on the EMG amplitude. Sleep stages were classified into REM sleep or NREM sleep according to the ratio of theta waves (4.0–10 Hz) or the density of delta waves (0.75–4.0 Hz), or EMG amplitude [33]. For each mouse, the duration of Wake, REM sleep, and NREM sleep was calculated over 1 h, 6 h, and 12 h intervals. Frequency analysis for each sleep stage was performed using SleepSign. EEG signals were analyzed using fast Fourier transform analysis in the 0–20 Hz range. The EEG spectral power at each frequency was normalized to the total power across the 0–20 Hz range within each sleep stage. The NREM delta power was calculated as the area under the curve (AUC) of the delta wave in the EEG power spectrum.

### 2.7 Statistics

All statistical analyses were performed using SAS Software for Windows, Release 9.4 (SAS Institute Japan). GraphPad Prism 10.03 was utilized to visualize the data graphically. The results are expressed as the mean with the corresponding standard error of the mean (mean ± SE). For sleep architecture and delta power, data were analyzed using an analysis of variance for a randomized block design, with treatment as a fixed effect and animal ID as a blocking factor. No treatment × block interaction term was included, in accordance with the standard randomized block design. The primary comparisons of interest were pairwise contrasts between each active treatment group and the vehicle group, which were evaluated using Dunnett's multiple comparisons test based on this randomized block ANOVA model. A two‐sided *p*‐value of less than 0.05 was considered statistically significant.

## 3 Results

### 3.1 Paroxetine and Sertraline (SSRIs) Decrease REM Sleep

**FIGURE 1:**
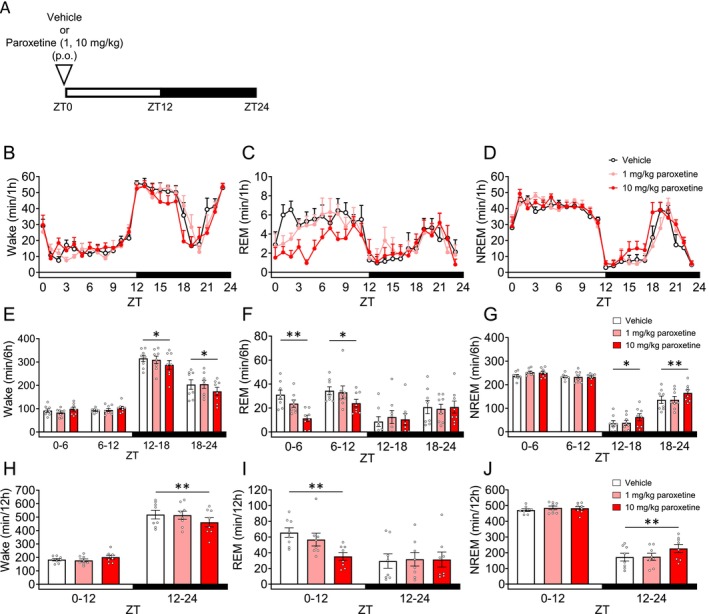
Effects of paroxetine on sleep architecture in mice. Each drug or vehicle was administered orally 30 min before ZT0. (A) Experimental Scheme. (B–D) Hourly durations in Wake, REM sleep and NREM sleep over a 24 h period. (E–G) Each 6 h duration in Wake, REM sleep and NREM sleep over a 24 h period. (H–J) Each 12 h duration in Wake, REM sleep and NREM sleep over a 24 h period. Data are expressed as mean ± SEM (*N* = 8). Statistical analyses were performed using a randomized block ANOVA (with animal ID as a blocking factor) followed by Dunnett's test. *p < 0.05, **p < 0.01 vs. Vehicle (E–J). Statistics are reported in Table S1. The white and black bars at the bottom of the graphs indicate the light (ZT0–12) and dark (ZT12–24) periods, respectively.

**FIGURE 2:**
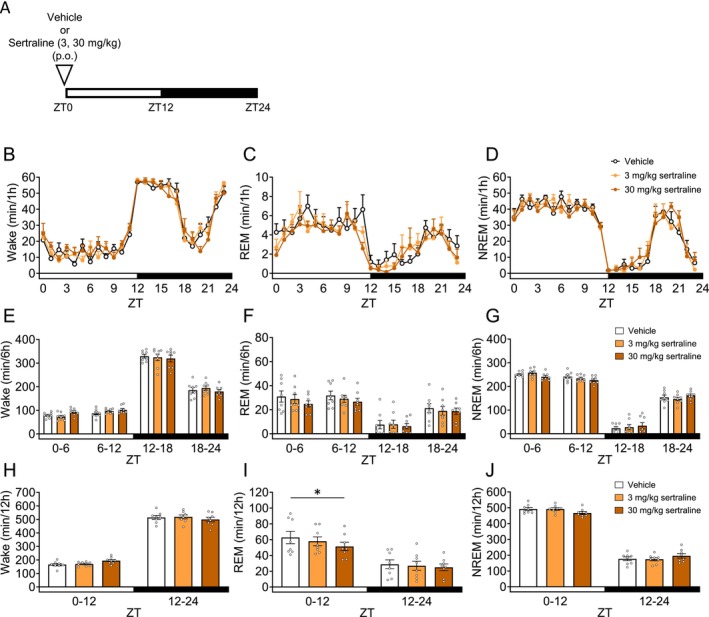
Effects of sertraline on sleep architecture in mice. Each drug or vehicle was administered orally 30 min before ZT0. (A) Experimental Scheme. (B–D) Hourly durations in Wake, REM sleep and NREM sleep over a 24 h period. (E–G) Each 6 h duration in Wake, REM sleep and NREM sleep over a 24 h period. (H–J) Each 12 h duration in Wake, REM sleep and NREM sleep over a 24 h period. Data are expressed as mean ± SEM (*N* = 8). Statistical analyses were performed using a randomized block ANOVA (with animal ID as a blocking factor) followed by Dunnett's test. *p < 0.05, **p < 0.01 vs. Vehicle (E–J). Statistics are reported in Table S1. The white and black bars at the bottom of the graphs indicate the light (ZT0–12) and dark (ZT12–24) periods, respectively.

Given that SSRIs have been reported to increase REM latency and suppress REM sleep, we investigated the effects of paroxetine and sertraline on sleep architecture [35]. First, we evaluated the effect of paroxetine. Mice were orally administered paroxetine 30 min before ZT0 (Figure 1A). In ZT0–6, ZT6–12 and ZT0–12, 10 mg/kg paroxetine significantly decreased REM sleep (Figure 1C,F,I, Table S1). Additionally, in ZT12–18, ZT18–24 and ZT12–24, 10 mg/kg paroxetine significantly decreased Wake (Figure 1B,E,H, Table S1), and significantly increased NREM sleep (Figure 1D,G,J, Table S1). Based on pharmacokinetic considerations, we conducted spectral power analysis in Wake/REM/NREM in ZT0–6 (Figure S1A–C). In NREM sleep, 10 mg/kg paroxetine significantly increased delta power (Figure S1D, Table S2). Next, we evaluated sertraline. Mice were orally administered sertraline 30 min before ZT0 (Figure 2A). In ZT0–12, 30 mg/kg sertraline significantly decreased REM sleep (Figure 2C,F,I, Table S1). In contrast, sertraline did not affect Wake (Figure 2B,E,H, Table S1) and NREM sleep (Figure 2D,G,J, Table S1). We conducted spectral power analysis in Wake/REM/NREM in ZT0–6 (Figure S2A–C). In NREM sleep, 30 mg/kg sertraline significantly increased delta power (Figure S2D, Table S2).

### 3.2 Duloxetine (SNRI) Increases NREM Sleep and Decreases REM Sleep

**FIGURE 3:**
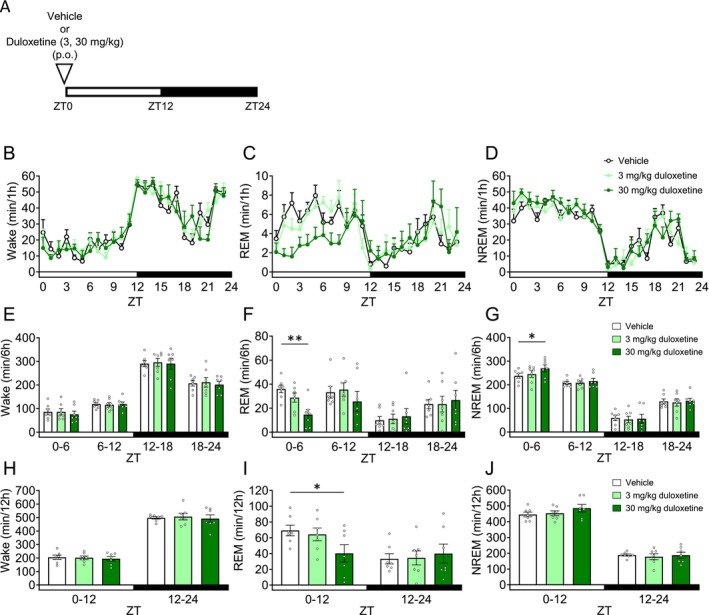
Effects of duloxetine on sleep architecture in mice. Each drug or vehicle was administered orally 30 min before ZT0. (A) Experimental Scheme. (B–D) Hourly durations in Wake, REM sleep and NREM sleep over a 24 h period. (E–G) Each 6 h duration in Wake, REM sleep and NREM sleep over a 24 h period. (H–J) Each 12 h duration in Wake, REM sleep and NREM sleep over a 24 h period. Data are expressed as mean ± SEM (*N* = 7). Statistical analyses were performed using a randomized block ANOVA (with animal ID as a blocking factor) followed by Dunnett's test. *p < 0.05, **p < 0.01 vs. Vehicle (E–J). Statistics are reported in Table S1. The white and black bars at the bottom of the graphs indicate the light (ZT0–12) and dark (ZT12–24) periods, respectively.

In healthy volunteers and patients with MDD, duloxetine reduced REM sleep [36, 37]. We evaluated the effect of duloxetine on sleep architecture. Mice were orally administered duloxetine 30 min before ZT0 (Figure 3A). In ZT0–6 and ZT0–12, 30 mg/kg duloxetine significantly decreased REM sleep (Figure 3C,F,I, Table S1). Moreover, while 30 mg/kg duloxetine significantly increased NREM sleep in ZT0–6 (Figure 3D,G,J, Table S1), Wake was not affected (Figure 3B,E,H, Table S1). We conducted spectral power analysis in Wake/REM/NREM in ZT0–6 (Figure S3A–C). In NREM sleep, 30 mg/kg duloxetine significantly increased delta power (Figure S3D, Table S2).

### 3.3 Mirtazapine (NaSSA) Decreases Wake and Increases NREM Sleep

**FIGURE 4:**
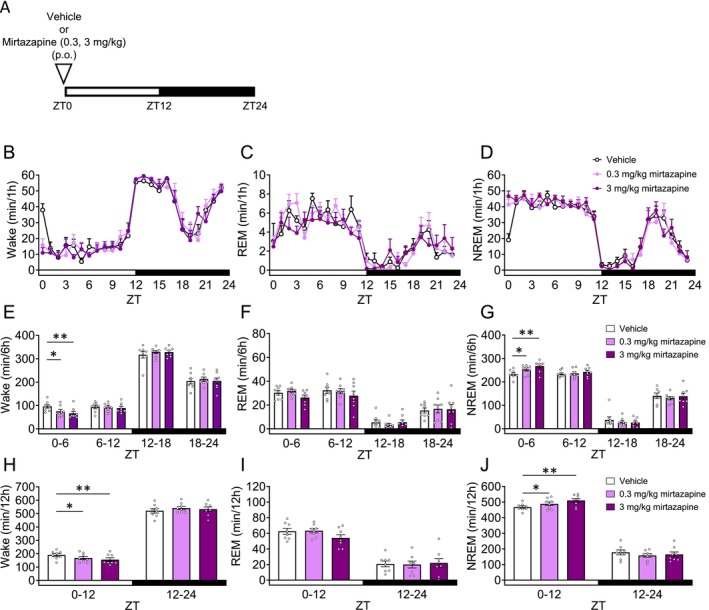
Effects of mirtazapine on sleep architecture in mice. Each drug or vehicle was administered orally 30 min before ZT0. (A) Experimental Scheme. (B–D) Hourly durations in Wake, REM sleep and NREM sleep over a 24 h period. (E–G) Each 6 h duration in Wake, REM sleep and NREM sleep over a 24 h period. (H–J) Each 12 h duration in Wake, REM sleep and NREM sleep over a 24 h period. Data are expressed as mean ± SEM (*N* = 8). Statistical analyses were performed using a randomized block ANOVA (with animal ID as a blocking factor) followed by Dunnett's test. *p < 0.05, **p < 0.01 vs. Vehicle (E–J). Statistics are reported in Table S1. The white and black bars at the bottom of the graphs indicate the light (ZT0–12) and dark (ZT12–24) periods, respectively.

Mirtazapine enhances the quality and duration of sleep in healthy volunteers and MDD patients [38, 39, 40]. We evaluated the effect of mirtazapine on sleep architecture. Mice were orally administered mirtazapine 30 min before ZT0 (Figure 4A). In ZT0–6 and ZT0–12, mirtazapine at doses of 0.3 and 3 mg/kg significantly decreased Wake (Figure 4B,E,H, Table S1), and significantly increased NREM sleep (Figure 4D,G,J, Table S1). On the other hand, mirtazapine did not affect REM sleep (Figure 4C,F,I, Table S1). We conducted spectral power analysis in Wake/REM/NREM in ZT0–6 (Figure S4A–C). In NREM sleep, 3 mg/kg mirtazapine significantly increased delta power (Figure S4D, Table S2).

### 3.4 Vortioxetine (S‐RIM) Does Not Affect REM Sleep

**FIGURE 5:**
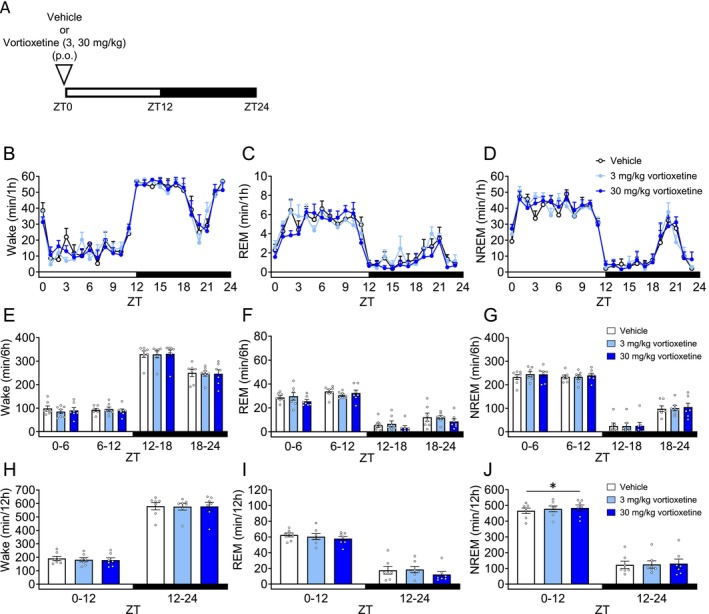
Effects of vortioxetine on sleep architecture in mice. Each drug or vehicle was administered orally 30 min before ZT0. (A) Experimental Scheme. (B–D) Hourly durations in Wake, REM sleep and NREM sleep over a 24 h period. (E–G) Each 6 h duration in Wake, REM sleep and NREM sleep over a 24 h period. (H–J) Each 12 h duration in Wake, REM sleep and NREM sleep over a 24 h period. Data are expressed as mean ± SEM (*N* = 7). Statistical analyses were performed using a randomized block ANOVA (with animal ID as a blocking factor) followed by Dunnett's test. *p < 0.05, **p < 0.01 vs. Vehicle (E–J). Statistics are reported in Table S1. The white and black bars at the bottom of the graphs indicate the light (ZT0–12) and dark (ZT12–24) periods, respectively.

In patients with MDD comorbid with sleep disturbance, some antidepressants can adversely affect sleep quality and continuity [35, 41]. Vortioxetine improves not only depression but also sleep disturbance in patients with MDD [42]. We evaluated the effect of vortioxetine on sleep architecture. Mice were orally administered vortioxetine 30 min before ZT0 (Figure 5A). In ZT0–12, 30 mg/kg vortioxetine significantly increased NREM sleep (Figure 5D,G,J, Table S1). Meanwhile, vortioxetine at doses of 3 and 30 mg/kg did not result in significant changes in Wake and REM sleep (Figure 5B,C,E,F,H,I, Table S1). We conducted spectral power analysis in Wake/REM/NREM in ZT0–6 (Figure S5A–C). Vortioxetine did not affect delta power (Figure S5D, Table S2).

## 4 Discussion

Because patients with MDD often experience sleep disturbances, it is necessary to consider the effects of pharmacological treatments on sleep architecture [9, 10, 11, 12, 13]. In the present study, we recorded EEGs from the cortex and EMGs from the trapezius muscle to evaluate the effects of five different antidepressants—paroxetine, sertraline, duloxetine, mirtazapine, and vortioxetine—on sleep architecture. To prevent damage to the EEG/EMG implants, mice were housed individually, although we recognize this isolation as a potential stressor. Therefore, running wheels were provided to ensure a robust circadian rhythm, and they also served as environmental enrichment to mitigate isolation stress [43, 44]. The doses of these drugs were selected based on previously reported doses at which central pharmacological effects were observed in rodents. Paroxetine, sertraline, duloxetine, mirtazapine, and vortioxetine improve depression‐ and anxiety‐like behaviors [22, 29, 30, 31, 45, 46, 47, 48]. In this study, significant effects on sleep architecture were generally observed at the higher doses tested for each compound. Therefore, the discussion focuses mainly on the results from these effective doses. Among the antidepressants tested, distinct effects on sleep architecture were observed. The SSRIs paroxetine and sertraline both reduced REM sleep, while the SNRI duloxetine not only decreased REM sleep but also increased NREM sleep. The NaSSA mirtazapine reduced Wake and increased NREM sleep, and the S‐RIM vortioxetine increased NREM sleep without affecting REM sleep.

In a clinical study, both paroxetine and sertraline were reported to reduce REM sleep and similar effects were also observed in mice (Figures 1, 2) [49, 50]. Although both drugs are classified as SSRIs, their effects on sleep architecture were different. While direct statistical comparison was not performed, paroxetine markedly suppressed REM sleep across multiple time points, whereas the effect of sertraline was more limited. This difference may be attributable to their respective pharmacological activities. Notably, mice lacking the muscarinic acetylcholine receptors *Chrm1* and *Chrm3* exhibited shortened REM sleep, suggesting that paroxetine's antagonistic activity on muscarinic acetylcholine receptors may contribute to its REM sleep‐suppressing properties [51, 52]. In contrast, sertraline is not expected to block muscarinic receptors in vivo due to its very low affinity, which may explain its relatively modest impact on sleep architecture in mice [53]. In patients, sertraline has been associated with a lower risk of sleep‐related adverse effects, such as somnolence and insomnia, compared to paroxetine [50]. Furthermore, nightmares following an increase in the dose of paroxetine have been reported, which subsequently improved after switching to sertraline [54, 55]. This report suggests that sertraline may exert antidepressant effects without disrupting sleep architecture.

Among the drugs evaluated in this study, duloxetine is the only agent approved for the treatment of pain‐related conditions, including fibromyalgia, diabetic peripheral neuropathy, and chronic musculoskeletal pain [56]. Duloxetine exerts analgesic effects by enhancing the activity of noradrenergic and serotonergic neurons in the descending spinal pathways that project to the dorsal horn, thereby modulating both neuropathic and chronic pain states [57]. In the present study, duloxetine increased NREM sleep and reduced REM sleep in mice (Figure 3). It has been reported that extracellular noradrenaline (NA) levels in the thalamus decrease during REM sleep and increase during NREM sleep in rodent models [18, 58]. Therefore, the changes in sleep architecture may be attributed to the simultaneous elevation of both 5‐HT and NA by duloxetine. In healthy volunteers, it has been reported that duloxetine prolonged REM latency, reduced REM sleep, and shortened sleep onset latency [36]. Furthermore, in patients with MDD, duloxetine significantly increased NREM stage 3 sleep while reducing REM latency and REM sleep duration [37]. In the present study, delta wave power during NREM sleep in the ZT0–6 period was markedly increased by 30 mg/kg duloxetine (Figure S3). Since delta waves are typically enhanced during deep NREM sleep, this result may partially reflect the clinical findings described above.

Mirtazapine increased NREM sleep without affecting REM sleep in mice (Figure 4). In preclinical studies, both histamine H_1_ receptor antagonists and 5‐HT_2A_ receptor antagonists were shown to increase NREM sleep. Therefore, mirtazapine may promote sleep through its antagonistic activity at the histamine H_1_ receptor and 5‐HT_2A_ receptor [19, 59, 60]. Similar findings have been reported in clinical studies, where mirtazapine shortened sleep onset latency and improved total sleep time and sleep efficiency in both depressive patients and healthy volunteers, while leaving REM sleep unchanged [38, 61]. These results suggest that the findings accurately reflect the unique profile of mirtazapine as an antidepressant that does not cause sleep disturbances. Notably, among the five antidepressants tested in this study, only mirtazapine significantly reduced Wake during the light phase (inactive phase), and these effects did not persist into the dark phase (active phase). Since the effect was maximal immediately after administration and diminished over time, this profile suggests that the increase in NREM sleep is driven by the direct pharmacological action of mirtazapine, which subsides as plasma concentrations decrease.

Vortioxetine significantly increased NREM sleep without affecting REM sleep in mice (Figure 5). However, the magnitude of this NREM sleep increase was very small compared to that observed with duloxetine or mirtazapine. Vortioxetine suppresses depression‐like and anxiety‐like behaviors and improves cognitive impairments in rodents [22]. The observation that vortioxetine did not affect REM sleep is partially consistent with findings from a previous study, in which a single administration of vortioxetine reduced REM sleep, but this effect disappeared in rats after three consecutive days of treatment [62]. Clinical studies showed that vortioxetine's influence on sleep architecture is relatively small, with reports indicating that it does not cause sleep disturbances in healthy volunteers unless administered at high doses [63, 64]. In contrast, it has been reported that SSRIs and SNRIs were associated with a significantly higher incidence of sleep‐related adverse events compared to placebo [65, 66]. It has been reported that 5‐HT_3_ receptor agonists suppress REM sleep in rodents and prolong REM sleep latency in healthy volunteers [67, 68]. Previous studies in rats showed that vortioxetine reduced REM sleep acutely, but this effect disappeared after repeated treatment [62]. In this study, vortioxetine did not suppress REM sleep in mice. This discrepancy is likely explained by species‐specific differences in 5‐HT_3_ receptor pharmacology. A recent structural analysis demonstrated that vortioxetine acts as a partial agonist at the human 5‐HT_3_ receptor but functions as an antagonist at the rodent receptor, due to a single amino acid difference [69]. Therefore, in mice, the antagonistic activity of vortioxetine at 5‐HT_3_ receptors likely counteracts the REM‐suppressing effects of increased synaptic serotonin, whereas in humans, its partial agonism may contribute to REM suppression. Taken together, these observations suggest that vortioxetine is a drug with minimal influence on sleep architecture in mice.

Regarding NREM delta power, duloxetine and mirtazapine increased both NREM sleep and delta power. Paroxetine and sertraline increased delta power but did not extend NREM sleep. Notably, among the tested drugs, only mirtazapine significantly reduced Wake during ZT0–12, indicating its strong sleep‐promoting profile.

This is the first report to demonstrate that paroxetine increases NREM sleep during ZT12–24 (the dark period) even when administered prior to ZT0. While previous studies have focused primarily on immediate sleep effects, our continuous monitoring revealed this delayed effect during the active phase. Previous studies have shown that paroxetine reduces locomotor activity during the dark period, while having no effect during the light period [70]. Furthermore, when administered immediately before the dark period, paroxetine increased sleep only during the dark period [71, 72]. These findings suggest that, among the antidepressants examined, only paroxetine has the potential to enhance NREM sleep specifically during the dark period. This effect may relate to reports of daytime drowsiness in MDD patients treated with paroxetine, as the dark period in mice represents the active phase, similar to daytime in humans. In clinical trials, paroxetine has been the SSRI most frequently associated with drowsiness, and patients treated with paroxetine often experience daytime sleepiness, whereas such effects are not observed in those treated with sertraline [73]. It is important to consider the pharmacokinetic profiles of these drugs. Reported oral half‐lives of these antidepressants in rodents are relatively short, ranging from approximately 2 to 6 h [74, 75, 76, 77]. Notably, although the half‐life of sertraline is reported to be longer than that of paroxetine in mice, only paroxetine induced changes in sleep architecture during the subsequent dark period (ZT12–24). This suggests that the delayed increase in NREM sleep observed with paroxetine is unlikely to be due to residual drug effects. Instead, similar to the delayed effects reported for zolpidem [78], this may reflect a rebound‐like or homeostatic compensatory response to the potent REM sleep suppression during the light period.

**FIGURE 6:**
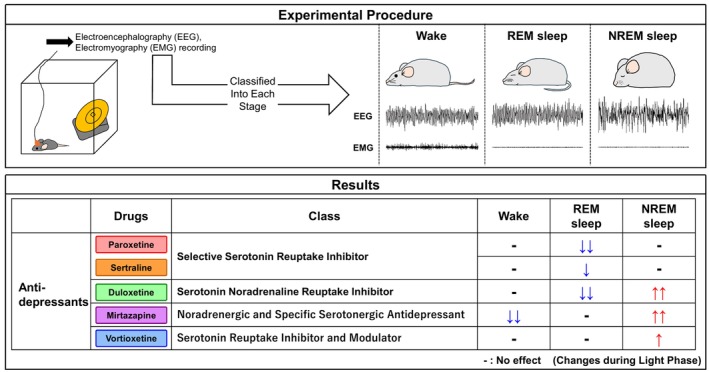
Overview of the differential effects of antidepressants on sleep architecture in mice. This schematic summary illustrates the changes in Wake, REM, and NREM sleep during the light phase (ZT0–12) following the administration of five distinct antidepressants.

This study is the first report comparing the effects of paroxetine, sertraline, duloxetine, mirtazapine, and vortioxetine on sleep architecture in mice under identical experimental conditions (Figure 6). While some of our findings confirm individual reports from previous literature, examining them together eliminates inter‐study variability and provides a reliable reference. Although drugs that do not affect sleep architecture may be considered advantageous in terms of minimizing sleep‐related adverse effects, a reduction in REM sleep may also play a significant role in the therapeutic efficacy of antidepressants. Notably, the three drugs in this study that have pronounced effects on sleep architecture—paroxetine, duloxetine, and mirtazapine—have all been reported to exhibit high antidepressant efficacy (response rates) [79]. On the other hand, excessive modulation of sleep architecture may lead to sleep‐related side effects and reduced medication adherence. Therefore, it is likely that selecting appropriate antidepressants based on individual patient conditions is essential and understanding each drug's impact on sleep architecture may be important in determining treatment strategies for MDD.

In the present study, drugs were administered orally 30 min prior to the light phase to evaluate their effects on sleep architecture during the resting period. However, administration prior to the dark phase (active period) may yield different pharmacological outcomes due to differences in baseline arousal levels. Furthermore, while we utilized oral administration, other routes could result in different pharmacokinetic profiles. This study was conducted using naïve male C57BL/6J mice housed in cages equipped with running wheels, and we evaluated the pharmacological characteristics of each compound. Therefore, the effects on sleep architecture may differ from the results in depressive animal models. In such models—including water immersion and restraint stress and chronic social defeat stress—abnormalities in sleep architecture have been reported [80, 81]. Evaluating antidepressants using these animal models may reveal effects that are more relevant to clinical studies. Similarly, further research is warranted to elucidate potential sex‐dependent effects, as ovarian hormones are known to modulate the serotonergic system and influence sleep architecture [82, 83]. Additionally, since mice are nocturnal animals, the results obtained here may not be directly extrapolated to human beings, who are diurnal. Furthermore, latency to sleep onset and REM sleep were not evaluated in this study. In naïve mice with polyphasic sleep patterns, these latency parameters can be highly variable compared to total sleep duration. Therefore, we focused on analyzing the duration and distribution of sleep stages to establish robust pharmacological profiles. Nevertheless, these findings may provide valuable insights when selecting a different antidepressant for individuals where sleep disturbances emerge during treatment.

## Author Contributions

**Junya Maruoka:** conceptualization, methodology, data curation, supervision, formal analysis, validation, investigation, visualization, project administration, writing – review and editing, writing – original draft. **Ryo Egami:** conceptualization, methodology, investigation, validation, writing – review and editing. **Yuto Akita:** conceptualization, investigation, methodology, validation, writing – review and editing. **Kohei Kozuka:** investigation, conceptualization, validation, methodology, writing – review and editing. **Yusuke Kakumoto:** methodology, formal analysis, writing – review and editing. **Tetsuro Kikuchi:** conceptualization, writing – review and editing, resources, project administration, supervision. **Kazuhiko Kume:** conceptualization, writing – review and editing, project administration, resources, supervision.

## Funding

This study was supported by Otsuka Pharmaceutical Co. Ltd. (Tokyo, Japan).

## Conflicts of Interest

J. Maruoka, Y. Kakumoto and T. Kikuchi are employees of Otsuka Pharmaceutical Co. Ltd. and own stock or stock options. The remaining authors have no actual or perceived conflicts of interest related to the contents of this article.

## Supporting information


**Figure S1:** Effects of paroxetine on normalized power in ZT0–6.
**Figure S2:** Effects of sertraline on normalized power in ZT0–6.
**Figure S3:** Effects of duloxetine on normalized power in ZT0–6.
**Figure S4:** Effects of mirtazapine on normalized power in ZT0–6.
**Figure S5:** Effects of vortioxetine on normalized power in ZT0–6.
**Table S1:** Statistics for sleep architecture in ZT0–24.
**Table S2:** Statistics for NREM delta power in ZT0–6.

## Data Availability

The data that supports the findings of this study are available in the supporting information of this article.
