# Supplementary material for: Comparative Sleep Architecture Profiles of Antidepressants in Mice: Pharmacological Characterization of Paroxetine, Sertraline, Duloxetine, Mirtazapine and Vortioxetine
Source: Pharmacol Res Perspect. 2026 May 4;14(3):e70246. doi: 10.1002/prp2.70246 (PMC13139623; doi:10.1002/prp2.70246)
Supplement: Supplementary file 1 — Figure S1: Effects of paroxetine on normalized power in ZT0–6. Figure S2: Effects of sertraline on normalized power in ZT0–6. Figure S3: Effects of duloxetine on normalized power in ZT0–6. Figure S4: Effects of mirtazapine on normalized power in ZT0–6. Figure S5: Effects of vortioxetine on normalized power in ZT0–6. Table S1: Statistics for sleep architecture in ZT0–24. Table S2: Statistics for NREM delta power in ZT0–6. [file PRP2-14-e70246-s001.docx]

**Comparative Sleep Architecture Profiles of Antidepressants in Mice: Pharmacological Characterization of Paroxetine, Sertraline, Duloxetine, Mirtazapine and Vortioxetine**

**Junya Maruoka, Ryo Egami, Yuto Akita, Kohei Kozuka, Yusuke Kakumoto, Tetsuro Kikuchi, Kazuhiko Kume.**

**Pharmacology Research & Perspectives**

**This Supplemental Data file includes:**

**Supplemental Figures 1 to 5**

**Supplemental Tables 1 to 2**

A

B

C

D

**

**Supplemental Figure 1. Effects of paroxetine on normalized power in ZT0–6.** Each drug or vehicle was administered orally 30 minutes before ZT0. A–C: Normalized power of mice during Wake, REM sleep and NREM sleep. D: The AUC of the delta wave (0.75–4 Hz) of the normalized power was measured during NREM sleep. Data are expressed as mean ± SEM (N=8). Statistical analyses were performed using a randomized block ANOVA (with animal ID as a blocking factor) followed by Dunnett’s test. *p<0.05, **p<0.01 vs. Vehicle (D) Statistics reported in Supplemental Table 2.

C

B

A

D

**

**Supplemental Figure 2. Effects of sertraline on normalized power in ZT0–6.** Each drug or vehicle was administered orally 30 minutes before ZT0. A–C: Normalized power of mice during Wake, REM sleep and NREM sleep. D: The AUC of the delta wave (0.75–4 Hz) of the normalized power was measured during NREM sleep. Data are expressed as mean ± SEM (N=8). Statistical analyses were performed using a randomized block ANOVA (with animal ID as a blocking factor) followed by Dunnett’s test. *p<0.05, **p<0.01 vs. Vehicle (D) Statistics reported in Supplemental Table 2.

A

B

C

D

**

**Supplemental Figure 3. Effects of duloxetine on normalized power in ZT0–6.** Each drug or vehicle was administered orally 30 minutes before ZT0. A–C: Normalized power of mice during Wake, REM sleep and NREM sleep. D: The AUC of the delta wave (0.75–4 Hz) of the normalized power was measured during NREM sleep. Data are expressed as mean ± SEM (N=7). Statistical analyses were performed using a randomized block ANOVA (with animal ID as a blocking factor) followed by Dunnett’s test. *p<0.05, **p<0.01 vs. Vehicle (D) Statistics reported in Supplemental Table 2.

C

B

A

D

**

**Supplemental Figure 4. Effects of mirtazapine on normalized power in ZT0–6.** Each drug or vehicle was administered orally 30 minutes before ZT0. A–C: Normalized power of mice during Wake, REM sleep and NREM sleep. D: The AUC of the delta wave (0.75–4 Hz) of the normalized power was measured during NREM sleep. Data are expressed as mean ± SEM (N=8). Statistical analyses were performed using a randomized block ANOVA (with animal ID as a blocking factor) followed by Dunnett’s test. *p<0.05, **p<0.01 vs. Vehicle (D) Statistics reported in Supplemental Table 2.

C

B

A

D

**Supplemental Figure 5. Effects of vortioxetine on normalized power in ZT0–6.** Each drug or vehicle was administered orally 30 minutes before ZT0. A–C: Normalized power of mice during Wake, REM sleep and NREM sleep. D: The AUC of the delta wave (0.75–4 Hz) of the normalized power was measured during NREM sleep. Data are expressed as mean ± SEM (N=7). Statistical analyses were performed using a randomized block ANOVA (with animal ID as a blocking factor) followed by Dunnett’s test. *p<0.05, **p<0.01 vs. Vehicle (D) Statistics reported in Supplemental Table 2.

**Supplemental Table 1. Statistics for sleep architecture in ZT0–24.** Paroxetine, sertraline, duloxetine, mirtazapine, vortioxetine and vehicle were orally administered. Statistical analyses were performed using a randomized block ANOVA (with animal ID as a blocking factor) followed by Dunnett’s test. vs. Vehicle

|  |  |  | Group | | Time (min) | | | P value |  | Group | | Time (min) | | | P value |
| --- | --- | --- | --- | --- | --- | --- | --- | --- | --- | --- | --- | --- | --- | --- | --- |
|  |  |  |  |  | Mean | ± | SE |  |  |  |  | Mean | ± | SE |  |
| **Paroxetine (N=8)** | Wake | ZT0–6 | Vehicle | | 91.5 | ± | 7.4 | _ | ZT0–12 | Vehicle | | 183.4 | ± | 8.6 | _ |
|  |  |  | 1 mg/kg | paroxetine | 83.5 | ± | 6.0 | 0.3691 |  | 1 mg/kg | paroxetine | 177.7 | ± | 11.3 | 0.7875 |
|  |  |  | 10 mg/kg | paroxetine | 98.3 | ± | 8.2 | 0.4791 |  | 10 mg/kg | paroxetine | 201.6 | ± | 14.4 | 0.1443 |
|  |  | ZT6–12 | Vehicle | | 91.8 | ± | 3.3 | _ |  |  |  |  |  |  |  |
|  |  |  | 1 mg/kg | paroxetine | 94.2 | ± | 6.8 | 0.8996 |  |  |  |  |  |  |  |
|  |  |  | 10 mg/kg | paroxetine | 103.3 | ± | 6.9 | 0.1526 |  |  |  |  |  |  |  |
|  |  | ZT12–18 | Vehicle | | 315.4 | ± | 13.8 | _ | ZT12–24 | Vehicle | | 518.7 | ± | 32.1 | _ |
|  |  |  | 1 mg/kg | paroxetine | 309.3 | ± | 16.1 | 0.7895 |  | 1 mg/kg | paroxetine | 514.0 | ± | 30.1 | 0.9366 |
|  |  |  | 10 mg/kg | paroxetine | 287.4 | ± | 19.1 | 0.0327 |  | 10 mg/kg | paroxetine | 461.6 | ± | 34.0 | 0.0052 |
|  |  | ZT18–24 | Vehicle | | 203.4 | ± | 20.3 | _ |  |  |  |  |  |  |  |
|  |  |  | 1 mg/kg | paroxetine | 204.7 | ± | 17.4 | 0.9845 |  |  |  |  |  |  |  |
|  |  |  | 10 mg/kg | paroxetine | 174.2 | ± | 16.9 | 0.0115 |  |  |  |  |  |  |  |
|  | REM | ZT0–6 | Vehicle | | 31.1 | ± | 3.8 | _ | ZT0–12 | Vehicle | | 65.6 | ± | 6.2 | _ |
|  |  |  | 1 mg/kg | paroxetine | 23.7 | ± | 3.0 | 0.0524 |  | 1 mg/kg | paroxetine | 56.8 | ± | 8.1 | 0.1659 |
|  |  |  | 10 mg/kg | paroxetine | 11.3 | ± | 2.7 | <0.0001 |  | 10 mg/kg | paroxetine | 35.3 | ± | 4.9 | <0.0001 |
|  |  | ZT6–12 | Vehicle | | 34.4 | ± | 3.3 | _ |  |  |  |  |  |  |  |
|  |  |  | 1 mg/kg | paroxetine | 33.1 | ± | 5.3 | 0.8966 |  |  |  |  |  |  |  |
|  |  |  | 10 mg/kg | paroxetine | 24.0 | ± | 3.1 | 0.0165 |  |  |  |  |  |  |  |
|  |  | ZT12–18 | Vehicle | | 8.7 | ± | 4.1 | _ | ZT12–24 | Vehicle | | 29.4 | ± | 9.1 | _ |
|  |  |  | 1 mg/kg | paroxetine | 12.5 | ± | 5.4 | 0.4047 |  | 1 mg/kg | paroxetine | 31.7 | ± | 8.6 | 0.7274 |
|  |  |  | 10 mg/kg | paroxetine | 10.5 | ± | 4.7 | 0.7906 |  | 10 mg/kg | paroxetine | 31.3 | ± | 9.6 | 0.7957 |
|  |  | ZT18–24 | Vehicle | | 20.7 | ± | 5.4 | _ |  |  |  |  |  |  |  |
|  |  |  | 1 mg/kg | paroxetine | 19.3 | ± | 4.0 | 0.7502 |  |  |  |  |  |  |  |
|  |  |  | 10 mg/kg | paroxetine | 20.8 | ± | 5.1 | 0.9989 |  |  |  |  |  |  |  |
|  | NREM | ZT0–6 | Vehicle | | 237.3 | ± | 6.0 | _ | ZT0–12 | Vehicle | | 471.1 | ± | 8.5 | _ |
|  |  |  | 1 mg/kg | paroxetine | 252.9 | ± | 4.7 | 0.0772 |  | 1 mg/kg | paroxetine | 485.6 | ± | 11.7 | 0.3854 |
|  |  |  | 10 mg/kg | paroxetine | 250.4 | ± | 6.9 | 0.1445 |  | 10 mg/kg | paroxetine | 483.1 | ± | 11.1 | 0.5057 |
|  |  | ZT6–12 | Vehicle | | 233.8 | ± | 4.4 | _ |  |  |  |  |  |  |  |
|  |  |  | 1 mg/kg | paroxetine | 232.7 | ± | 8.5 | 0.9820 |  |  |  |  |  |  |  |
|  |  |  | 10 mg/kg | paroxetine | 232.7 | ± | 5.5 | 0.9824 |  |  |  |  |  |  |  |
|  |  | ZT12–18 | Vehicle | | 35.9 | ± | 11.7 | _ | ZT12–24 | Vehicle | | 171.9 | ± | 24.7 | _ |
|  |  |  | 1 mg/kg | paroxetine | 38.2 | ± | 11.0 | 0.9585 |  | 1 mg/kg | paroxetine | 174.3 | ± | 22.6 | 0.9785 |
|  |  |  | 10 mg/kg | paroxetine | 62.1 | ± | 14.7 | 0.0285 |  | 10 mg/kg | paroxetine | 227.1 | ± | 25.6 | 0.0034 |
|  |  | ZT18–24 | Vehicle | | 135.9 | ± | 15.6 | _ |  |  |  |  |  |  |  |
|  |  |  | 1 mg/kg | paroxetine | 136.1 | ± | 14.2 | 0.9996 |  |  |  |  |  |  |  |
|  |  |  | 10 mg/kg | paroxetine | 165.0 | ± | 12.9 | 0.0048 |  |  |  |  |  |  |  |

|  |  |  | Group | | Time (min) | | | P value |  | Group | | Time (min) | | | P value |
| --- | --- | --- | --- | --- | --- | --- | --- | --- | --- | --- | --- | --- | --- | --- | --- |
|  |  |  |  |  | Mean | ± | SE |  |  |  |  | Mean | ± | SE |  |
| **Sertraline (N=8)** | Wake | ZT0–6 | Vehicle | | 78.6 | ± | 4.2 | _ | ZT0–12 | Vehicle | | 165.4 | ± | 8.5 | _ |
|  |  |  | 3 mg/kg | sertraline | 73.2 | ± | 5.6 | 0.6480 |  | 3 mg/kg | sertraline | 169.9 | ± | 5.6 | 0.9042 |
|  |  |  | 30 mg/kg | sertraline | 92.9 | ± | 5.4 | 0.0934 |  | 30 mg/kg | sertraline | 194.8 | ± | 8.7 | 0.0533 |
|  |  | ZT6–12 | Vehicle | | 86.8 | ± | 6.2 | _ |  |  |  |  |  |  |  |
|  |  |  | 3 mg/kg | sertraline | 96.7 | ± | 3.5 | 0.4312 |  |  |  |  |  |  |  |
|  |  |  | 30 mg/kg | sertraline | 101.9 | ± | 5.6 | 0.1773 |  |  |  |  |  |  |  |
|  |  | ZT12–18 | Vehicle | | 329.1 | ± | 8.0 | _ | ZT12–24 | Vehicle | | 514.3 | ± | 14.5 | _ |
|  |  |  | 3 mg/kg | sertraline | 324.5 | ± | 14.0 | 0.8824 |  | 3 mg/kg | sertraline | 518.7 | ± | 14.4 | 0.9454 |
|  |  |  | 30 mg/kg | sertraline | 319.6 | ± | 14.2 | 0.6095 |  | 30 mg/kg | sertraline | 499.3 | ± | 16.7 | 0.5494 |
|  |  | ZT18–24 | Vehicle | | 185.1 | ± | 11.3 | _ |  |  |  |  |  |  |  |
|  |  |  | 3 mg/kg | sertraline | 194.2 | ± | 9.2 | 0.6668 |  |  |  |  |  |  |  |
|  |  |  | 30 mg/kg | sertraline | 179.7 | ± | 8.2 | 0.8569 |  |  |  |  |  |  |  |
|  | REM | ZT0–6 | Vehicle | | 30.9 | ± | 4.6 | _ | ZT0–12 | Vehicle | | 62.8 | ± | 7.8 | _ |
|  |  |  | 3 mg/kg | sertraline | 28.9 | ± | 3.7 | 0.6891 |  | 3 mg/kg | sertraline | 58.0 | ± | 5.5 | 0.3367 |
|  |  |  | 30 mg/kg | sertraline | 24.8 | ± | 2.5 | 0.0753 |  | 30 mg/kg | sertraline | 51.4 | ± | 5.3 | 0.0134 |
|  |  | ZT6–12 | Vehicle | | 31.8 | ± | 3.6 | _ |  |  |  |  |  |  |  |
|  |  |  | 3 mg/kg | sertraline | 29.1 | ± | 3.0 | 0.3928 |  |  |  |  |  |  |  |
|  |  |  | 30 mg/kg | sertraline | 26.6 | ± | 2.9 | 0.0693 |  |  |  |  |  |  |  |
|  |  | ZT12–18 | Vehicle | | 7.5 | ± | 3.5 | _ | ZT12–24 | Vehicle | | 28.8 | ± | 5.7 | _ |
|  |  |  | 3 mg/kg | sertraline | 7.9 | ± | 3.4 | 0.9812 |  | 3 mg/kg | sertraline | 26.9 | ± | 5.8 | 0.8342 |
|  |  |  | 30 mg/kg | sertraline | 6.3 | ± | 2.3 | 0.7182 |  | 30 mg/kg | sertraline | 25.0 | ± | 4.3 | 0.5063 |
|  |  | ZT18–24 | Vehicle | | 21.2 | ± | 3.8 | _ |  |  |  |  |  |  |  |
|  |  |  | 3 mg/kg | sertraline | 19.0 | ± | 3.6 | 0.6544 |  |  |  |  |  |  |  |
|  |  |  | 30 mg/kg | sertraline | 18.7 | ± | 2.7 | 0.5846 |  |  |  |  |  |  |  |
|  | NREM | ZT0–6 | Vehicle | | 250.4 | ± | 4.2 | _ | ZT0–12 | Vehicle | | 491.8 | ± | 9.7 | _ |
|  |  |  | 3 mg/kg | sertraline | 257.9 | ± | 6.3 | 0.3806 |  | 3 mg/kg | sertraline | 492.1 | ± | 8.4 | 0.9996 |
|  |  |  | 30 mg/kg | sertraline | 240.5 | ± | 5.6 | 0.2032 |  | 30 mg/kg | sertraline | 466.9 | ± | 9.0 | 0.1068 |
|  |  | ZT6–12 | Vehicle | | 241.4 | ± | 6.8 | _ |  |  |  |  |  |  |  |
|  |  |  | 3 mg/kg | sertraline | 234.2 | ± | 4.8 | 0.6709 |  |  |  |  |  |  |  |
|  |  |  | 30 mg/kg | sertraline | 226.4 | ± | 5.9 | 0.2258 |  |  |  |  |  |  |  |
|  |  | ZT12–18 | Vehicle | | 23.3 | ± | 6.6 | _ | ZT12–24 | Vehicle | | 177.0 | ± | 12.1 | _ |
|  |  |  | 3 mg/kg | sertraline | 27.6 | ± | 10.8 | 0.8947 |  | 3 mg/kg | sertraline | 174.5 | ± | 9.9 | 0.9764 |
|  |  |  | 30 mg/kg | sertraline | 34.1 | ± | 12.9 | 0.5243 |  | 30 mg/kg | sertraline | 195.8 | ± | 14.3 | 0.3258 |
|  |  | ZT18–24 | Vehicle | | 153.6 | ± | 9.7 | _ |  |  |  |  |  |  |  |
|  |  |  | 3 mg/kg | sertraline | 146.8 | ± | 7.5 | 0.7419 |  |  |  |  |  |  |  |
|  |  |  | 30 mg/kg | sertraline | 161.6 | ± | 6.4 | 0.6689 |  |  |  |  |  |  |  |

|  |  |  | Group | | Time (min) | | | P value |  | Group | | Time (min) | | | P value |
| --- | --- | --- | --- | --- | --- | --- | --- | --- | --- | --- | --- | --- | --- | --- | --- |
|  |  |  |  |  | Mean | ± | SE |  |  |  |  | Mean | ± | SE |  |
| **Duloxetine (N=7)** | Wake | ZT0–6 | Vehicle | | 86.2 | ± | 12.1 | _ | ZT0–12 | Vehicle | | 205.4 | ± | 16.2 | _ |
|  |  |  | 3 mg/kg | duloxetine | 86.1 | ± | 13.3 | 0.9999 |  | 3 mg/kg | duloxetine | 202.3 | ± | 13.2 | 0.9595 |
|  |  |  | 30 mg/kg | duloxetine | 75.8 | ± | 13.0 | 0.1990 |  | 30 mg/kg | duloxetine | 194.5 | ± | 16.4 | 0.6304 |
|  |  | ZT6–12 | Vehicle | | 119.2 | ± | 6.5 | _ |  |  |  |  |  |  |  |
|  |  |  | 3 mg/kg | duloxetine | 116.2 | ± | 9.1 | 0.9238 |  |  |  |  |  |  |  |
|  |  |  | 30 mg/kg | duloxetine | 118.8 | ± | 9.4 | 0.9984 |  |  |  |  |  |  |  |
|  |  | ZT12–18 | Vehicle | | 290.6 | ± | 12.7 | _ | ZT12–24 | Vehicle | | 497.8 | ± | 9.2 | _ |
|  |  |  | 3 mg/kg | duloxetine | 295.8 | ± | 16.5 | 0.9301 |  | 3 mg/kg | duloxetine | 507.3 | ± | 24.5 | 0.8752 |
|  |  |  | 30 mg/kg | duloxetine | 290.5 | ± | 23.4 | 0.9999 |  | 30 mg/kg | duloxetine | 492.2 | ± | 27.9 | 0.9552 |
|  |  | ZT18–24 | Vehicle | | 207.2 | ± | 11.6 | _ |  |  |  |  |  |  |  |
|  |  |  | 3 mg/kg | duloxetine | 211.5 | ± | 20.4 | 0.9435 |  |  |  |  |  |  |  |
|  |  |  | 30 mg/kg | duloxetine | 201.8 | ± | 13.4 | 0.9133 |  |  |  |  |  |  |  |
|  | REM | ZT0–6 | Vehicle | | 36.0 | ± | 3.2 | _ | ZT0–12 | Vehicle | | 69.3 | ± | 6.7 | _ |
|  |  |  | 3 mg/kg | duloxetine | 28.8 | ± | 3.8 | 0.2191 |  | 3 mg/kg | duloxetine | 64.3 | ± | 8.1 | 0.8442 |
|  |  |  | 30 mg/kg | duloxetine | 14.6 | ± | 4.1 | 0.0008 |  | 30 mg/kg | duloxetine | 40.2 | ± | 11.0 | 0.0264 |
|  |  | ZT6–12 | Vehicle | | 33.2 | ± | 4.9 | _ |  |  |  |  |  |  |  |
|  |  |  | 3 mg/kg | duloxetine | 35.5 | ± | 5.5 | 0.9141 |  |  |  |  |  |  |  |
|  |  |  | 30 mg/kg | duloxetine | 25.6 | ± | 8.4 | 0.4050 |  |  |  |  |  |  |  |
|  |  | ZT12–18 | Vehicle | | 9.9 | ± | 3.2 | _ | ZT12–24 | Vehicle | | 33.3 | ± | 6.5 | _ |
|  |  |  | 3 mg/kg | duloxetine | 11.1 | ± | 4.0 | 0.9414 |  | 3 mg/kg | duloxetine | 34.4 | ± | 8.9 | 0.9850 |
|  |  |  | 30 mg/kg | duloxetine | 13.2 | ± | 6.4 | 0.6660 |  | 30 mg/kg | duloxetine | 39.9 | ± | 12.0 | 0.6321 |
|  |  | ZT18–24 | Vehicle | | 23.4 | ± | 3.8 | _ |  |  |  |  |  |  |  |
|  |  |  | 3 mg/kg | duloxetine | 23.3 | ± | 6.6 | 0.9998 |  |  |  |  |  |  |  |
|  |  |  | 30 mg/kg | duloxetine | 26.7 | ± | 8.0 | 0.8120 |  |  |  |  |  |  |  |
|  | NREM | ZT0–6 | Vehicle | | 237.8 | ± | 10.0 | _ | ZT0–12 | Vehicle | | 445.4 | ± | 15.1 | _ |
|  |  |  | 3 mg/kg | duloxetine | 245.1 | ± | 14.0 | 0.6757 |  | 3 mg/kg | duloxetine | 453.5 | ± | 15.3 | 0.8864 |
|  |  |  | 30 mg/kg | duloxetine | 269.6 | ± | 14.7 | 0.0115 |  | 30 mg/kg | duloxetine | 485.3 | ± | 24.3 | 0.1156 |
|  |  | ZT6–12 | Vehicle | | 207.5 | ± | 6.7 | _ |  |  |  |  |  |  |  |
|  |  |  | 3 mg/kg | duloxetine | 208.3 | ± | 8.0 | 0.9969 |  |  |  |  |  |  |  |
|  |  |  | 30 mg/kg | duloxetine | 215.6 | ± | 11.4 | 0.7390 |  |  |  |  |  |  |  |
|  |  | ZT12–18 | Vehicle | | 59.5 | ± | 11.4 | _ | ZT12–24 | Vehicle | | 189.0 | ± | 6.8 | _ |
|  |  |  | 3 mg/kg | duloxetine | 53.1 | ± | 13.9 | 0.8375 |  | 3 mg/kg | duloxetine | 178.3 | ± | 18.3 | 0.7814 |
|  |  |  | 30 mg/kg | duloxetine | 56.3 | ± | 18.2 | 0.9561 |  | 30 mg/kg | duloxetine | 187.9 | ± | 18.9 | 0.9971 |
|  |  | ZT18–24 | Vehicle | | 129.4 | ± | 10.7 | _ |  |  |  |  |  |  |  |
|  |  |  | 3 mg/kg | duloxetine | 125.2 | ± | 15.5 | 0.9365 |  |  |  |  |  |  |  |
|  |  |  | 30 mg/kg | duloxetine | 131.5 | ± | 10.9 | 0.9834 |  |  |  |  |  |  |  |

|  |  |  | Group | | Time (min) | | | P value |  | Group | | Time (min) | | | P value |
| --- | --- | --- | --- | --- | --- | --- | --- | --- | --- | --- | --- | --- | --- | --- | --- |
|  |  |  |  |  | Mean | ± | SE |  |  |  |  | Mean | ± | SE |  |
| **Mirtazapine (N=8)** | Wake | ZT0–6 | Vehicle | | 96.1 | ± | 7.5 | _ | ZT0–12 | Vehicle | | 189.9 | ± | 11.0 | _ |
|  |  |  | 0.3 mg/kg | mirtazapine | 75.1 | ± | 7.8 | 0.0128 |  | 0.3 mg/kg | mirtazapine | 167.4 | ± | 12.0 | 0.0274 |
|  |  |  | 3 mg/kg | mirtazapine | 66.4 | ± | 8.4 | 0.0010 |  | 3 mg/kg | mirtazapine | 155.7 | ± | 13.3 | 0.0017 |
|  |  | ZT6–12 | Vehicle | | 93.9 | ± | 7.2 | _ |  |  |  |  |  |  |  |
|  |  |  | 0.3 mg/kg | mirtazapine | 92.3 | ± | 5.9 | 0.9661 |  |  |  |  |  |  |  |
|  |  |  | 3 mg/kg | mirtazapine | 89.3 | ± | 7.2 | 0.7655 |  |  |  |  |  |  |  |
|  |  | ZT12–18 | Vehicle | | 317.0 | ± | 13.9 | _ | ZT12–24 | Vehicle | | 521.2 | ± | 16.8 | _ |
|  |  |  | 0.3 mg/kg | mirtazapine | 328.8 | ± | 7.0 | 0.3461 |  | 0.3 mg/kg | mirtazapine | 541.5 | ± | 12.8 | 0.2169 |
|  |  |  | 3 mg/kg | mirtazapine | 328.4 | ± | 6.9 | 0.3683 |  | 3 mg/kg | mirtazapine | 533.4 | ± | 17.1 | 0.5357 |
|  |  | ZT18–24 | Vehicle | | 204.1 | ± | 12.0 | _ |  |  |  |  |  |  |  |
|  |  |  | 0.3 mg/kg | mirtazapine | 212.7 | ± | 8.6 | 0.5899 |  |  |  |  |  |  |  |
|  |  |  | 3 mg/kg | mirtazapine | 205.0 | ± | 12.7 | 0.9945 |  |  |  |  |  |  |  |
|  | REM | ZT0–6 | Vehicle | | 30.2 | ± | 1.9 | _ | ZT0–12 | Vehicle | | 62.6 | ± | 3.8 | _ |
|  |  |  | 0.3 mg/kg | mirtazapine | 31.8 | ± | 1.5 | 0.7047 |  | 0.3 mg/kg | mirtazapine | 63.4 | ± | 2.8 | 0.9724 |
|  |  |  | 3 mg/kg | mirtazapine | 26.3 | ± | 2.1 | 0.1716 |  | 3 mg/kg | mirtazapine | 54.1 | ± | 4.2 | 0.0894 |
|  |  | ZT6–12 | Vehicle | | 32.4 | ± | 2.6 | _ |  |  |  |  |  |  |  |
|  |  |  | 0.3 mg/kg | mirtazapine | 31.6 | ± | 2.2 | 0.9545 |  |  |  |  |  |  |  |
|  |  |  | 3 mg/kg | mirtazapine | 27.9 | ± | 3.8 | 0.2971 |  |  |  |  |  |  |  |
|  |  | ZT12–18 | Vehicle | | 5.4 | ± | 2.1 | _ | ZT12–24 | Vehicle | | 20.8 | ± | 3.6 | _ |
|  |  |  | 0.3 mg/kg | mirtazapine | 3.3 | ± | 1.3 | 0.1374 |  | 0.3 mg/kg | mirtazapine | 20.0 | ± | 4.4 | 0.9289 |
|  |  |  | 3 mg/kg | mirtazapine | 5.5 | ± | 2.0 | 0.9942 |  | 3 mg/kg | mirtazapine | 22.0 | ± | 5.9 | 0.8605 |
|  |  | ZT18–24 | Vehicle | | 15.4 | ± | 2.1 | _ |  |  |  |  |  |  |  |
|  |  |  | 0.3 mg/kg | mirtazapine | 16.8 | ± | 3.3 | 0.7984 |  |  |  |  |  |  |  |
|  |  |  | 3 mg/kg | mirtazapine | 16.5 | ± | 3.9 | 0.8699 |  |  |  |  |  |  |  |
|  | NREM | ZT0–6 | Vehicle | | 233.7 | ± | 6.4 | _ | ZT0–12 | Vehicle | | 467.5 | ± | 8.2 | _ |
|  |  |  | 0.3 mg/kg | mirtazapine | 253.0 | ± | 6.9 | 0.0170 |  | 0.3 mg/kg | mirtazapine | 489.2 | ± | 11.9 | 0.0207 |
|  |  |  | 3 mg/kg | mirtazapine | 267.3 | ± | 8.5 | 0.0002 |  | 3 mg/kg | mirtazapine | 510.1 | ± | 12.8 | 0.0001 |
|  |  | ZT6–12 | Vehicle | | 233.8 | ± | 4.9 | _ |  |  |  |  |  |  |  |
|  |  |  | 0.3 mg/kg | mirtazapine | 236.2 | ± | 6.7 | 0.8828 |  |  |  |  |  |  |  |
|  |  |  | 3 mg/kg | mirtazapine | 242.8 | ± | 6.9 | 0.2363 |  |  |  |  |  |  |  |
|  |  | ZT12–18 | Vehicle | | 37.6 | ± | 13.0 | _ | ZT12–24 | Vehicle | | 178.0 | ± | 15.5 | _ |
|  |  |  | 0.3 mg/kg | mirtazapine | 27.9 | ± | 6.4 | 0.4515 |  | 0.3 mg/kg | mirtazapine | 158.5 | ± | 11.2 | 0.2248 |
|  |  |  | 3 mg/kg | mirtazapine | 26.1 | ± | 6.9 | 0.3381 |  | 3 mg/kg | mirtazapine | 164.6 | ± | 16.2 | 0.4625 |
|  |  | ZT18–24 | Vehicle | | 140.4 | ± | 11.1 | _ |  |  |  |  |  |  |  |
|  |  |  | 0.3 mg/kg | mirtazapine | 130.5 | ± | 7.3 | 0.4803 |  |  |  |  |  |  |  |
|  |  |  | 3 mg/kg | mirtazapine | 138.6 | ± | 12.2 | 0.9704 |  |  |  |  |  |  |  |

|  |  |  | Group | | Time (min) | | | P value |  | Group | | Time (min) | | | P value |
| --- | --- | --- | --- | --- | --- | --- | --- | --- | --- | --- | --- | --- | --- | --- | --- |
|  |  |  |  |  | Mean | ± | SE |  |  |  |  | Mean | ± | SE |  |
| **Vortioxetine (N=7)** | Wake | ZT0–6 | Vehicle | | 98.6 | ± | 10.6 | _ | ZT0–12 | Vehicle | | 191.4 | ± | 15.9 | _ |
|  |  |  | 3 mg/kg | vortioxetine | 85.4 | ± | 8.9 | 0.1473 |  | 3 mg/kg | vortioxetine | 181.5 | ± | 16.1 | 0.2034 |
|  |  |  | 30 mg/kg | vortioxetine | 90.4 | ± | 11.8 | 0.4213 |  | 30 mg/kg | vortioxetine | 178.4 | ± | 17.7 | 0.0864 |
|  |  | ZT6–12 | Vehicle | | 92.8 | ± | 6.4 | _ |  |  |  |  |  |  |  |
|  |  |  | 3 mg/kg | vortioxetine | 96.1 | ± | 8.8 | 0.7907 |  |  |  |  |  |  |  |
|  |  |  | 30 mg/kg | vortioxetine | 88.0 | ± | 8.3 | 0.6128 |  |  |  |  |  |  |  |
|  |  | ZT12–18 | Vehicle | | 329.9 | ± | 14.2 | _ | ZT12–24 | Vehicle | | 579.9 | ± | 27.3 | _ |
|  |  |  | 3 mg/kg | vortioxetine | 328.8 | ± | 14.6 | 0.9541 |  | 3 mg/kg | vortioxetine | 576.2 | ± | 25.2 | 0.9655 |
|  |  |  | 30 mg/kg | vortioxetine | 331.2 | ± | 16.5 | 0.9271 |  | 30 mg/kg | vortioxetine | 577.5 | ± | 31.7 | 0.9851 |
|  |  | ZT18–24 | Vehicle | | 250.0 | ± | 15.1 | _ |  |  |  |  |  |  |  |
|  |  |  | 3 mg/kg | vortioxetine | 247.4 | ± | 11.2 | 0.9740 |  |  |  |  |  |  |  |
|  |  |  | 30 mg/kg | vortioxetine | 246.3 | ± | 17.2 | 0.9488 |  |  |  |  |  |  |  |
|  | REM | ZT0–6 | Vehicle | | 28.7 | ± | 1.4 | _ | ZT0–12 | Vehicle | | 62.5 | ± | 2.6 | _ |
|  |  |  | 3 mg/kg | vortioxetine | 29.7 | ± | 3.1 | 0.8190 |  | 3 mg/kg | vortioxetine | 60.4 | ± | 3.9 | 0.4733 |
|  |  |  | 30 mg/kg | vortioxetine | 25.5 | ± | 1.5 | 0.1746 |  | 30 mg/kg | vortioxetine | 57.9 | ± | 3.0 | 0.0649 |
|  |  | ZT6–12 | Vehicle | | 33.8 | ± | 1.7 | _ |  |  |  |  |  |  |  |
|  |  |  | 3 mg/kg | vortioxetine | 30.7 | ± | 1.0 | 0.1755 |  |  |  |  |  |  |  |
|  |  |  | 30 mg/kg | vortioxetine | 32.4 | ± | 2.6 | 0.6517 |  |  |  |  |  |  |  |
|  |  | ZT12–18 | Vehicle | | 5.5 | ± | 1.9 | _ | ZT12–24 | Vehicle | | 17.6 | ± | 5.0 | _ |
|  |  |  | 3 mg/kg | vortioxetine | 6.6 | ± | 2.5 | 0.4765 |  | 3 mg/kg | vortioxetine | 18.6 | ± | 4.0 | 0.9203 |
|  |  |  | 30 mg/kg | vortioxetine | 3.5 | ± | 1.7 | 0.1899 |  | 30 mg/kg | vortioxetine | 12.2 | ± | 3.7 | 0.1430 |
|  |  | ZT18–24 | Vehicle | | 12.2 | ± | 3.5 | _ |  |  |  |  |  |  |  |
|  |  |  | 3 mg/kg | vortioxetine | 12.0 | ± | 1.6 | 0.9915 |  |  |  |  |  |  |  |
|  |  |  | 30 mg/kg | vortioxetine | 8.7 | ± | 2.2 | 0.2460 |  |  |  |  |  |  |  |
|  | NREM | ZT0–6 | Vehicle | | 232.6 | ± | 11.2 | _ | ZT0–12 | Vehicle | | 466.0 | ± | 17.6 | _ |
|  |  |  | 3 mg/kg | vortioxetine | 244.9 | ± | 10.2 | 0.1751 |  | 3 mg/kg | vortioxetine | 478.1 | ± | 18.6 | 0.1084 |
|  |  |  | 30 mg/kg | vortioxetine | 244.1 | ± | 11.9 | 0.2090 |  | 30 mg/kg | vortioxetine | 483.7 | ± | 19.4 | 0.0199 |
|  |  | ZT6–12 | Vehicle | | 233.4 | ± | 7.9 | _ |  |  |  |  |  |  |  |
|  |  |  | 3 mg/kg | vortioxetine | 233.3 | ± | 9.4 | 0.9995 |  |  |  |  |  |  |  |
|  |  |  | 30 mg/kg | vortioxetine | 239.6 | ± | 9.4 | 0.4518 |  |  |  |  |  |  |  |
|  |  | ZT12–18 | Vehicle | | 24.7 | ± | 12.7 | _ | ZT12–24 | Vehicle | | 122.5 | ± | 22.9 | _ |
|  |  |  | 3 mg/kg | vortioxetine | 24.5 | ± | 12.8 | 0.9992 |  | 3 mg/kg | vortioxetine | 125.2 | ± | 23.1 | 0.9817 |
|  |  |  | 30 mg/kg | vortioxetine | 25.3 | ± | 14.9 | 0.9823 |  | 30 mg/kg | vortioxetine | 130.3 | ± | 28.7 | 0.8587 |
|  |  | ZT18–24 | Vehicle | | 97.8 | ± | 12.1 | _ |  |  |  |  |  |  |  |
|  |  |  | 3 mg/kg | vortioxetine | 100.6 | ± | 10.9 | 0.9692 |  |  |  |  |  |  |  |
|  |  |  | 30 mg/kg | vortioxetine | 105.0 | ± | 16.1 | 0.8236 |  |  |  |  |  |  |  |

**Supplemental Table 2. Statistics for NREM delta power in ZT0–6.** Paroxetine, sertraline, duloxetine, mirtazapine, vortioxetine and vehicle were orally administered. Statistical analyses were performed using a randomized block ANOVA (with animal ID as a blocking factor) followed by Dunnett’s test. vs. Vehicle

| NREM delta power | Group | | Mean | ± | SE | P value |
| --- | --- | --- | --- | --- | --- | --- |
| **Paroxetine (N=8)** | Vehicle | | 11.61 | ± | 0.53 | _ |
|  | 1 mg/kg | paroxetine | 11.74 | ± | 0.57 | 0.7873 |
|  | 10 mg/kg | paroxetine | 12.43 | ± | 0.62 | 0.0042 |
| **Sertraline (N=8)** | Vehicle | | 11.90 | ± | 0.64 | _ |
|  | 3 mg/kg | sertraline | 11.93 | ± | 0.67 | 0.9541 |
|  | 30 mg/kg | sertraline | 12.41 | ± | 0.64 | 0.0047 |
| **Duloxetine (N=7)** | Vehicle | | 11.82 | ± | 0.50 | _ |
|  | 3 mg/kg | duloxetine | 12.06 | ± | 0.43 | 0.9096 |
|  | 30 mg/kg | duloxetine | 14.29 | ± | 0.80 | 0.0056 |
| **Mirtazapine (N=8)** | Vehicle | | 12.24 | ± | 0.36 | _ |
|  | 0.3 mg/kg | mirtazapine | 12.54 | ± | 0.37 | 0.4478 |
|  | 3 mg/kg | mirtazapine | 13.38 | ± | 0.37 | 0.0014 |
| **Vortioxetine (N=7)** | Vehicle | | 12.62 | ± | 0.64 | _ |
|  | 3 mg/kg | vortioxetine | 12.85 | ± | 0.72 | 0.4362 |
|  | 30 mg/kg | vortioxetine | 13.04 | ± | 0.68 | 0.0983 |
